# Neuropeptide S-Mediated Facilitation of Synaptic Transmission Enforces Subthreshold Theta Oscillations within the Lateral Amygdala

**DOI:** 10.1371/journal.pone.0018020

**Published:** 2011-03-18

**Authors:** Susanne Meis, Oliver Stork, Thomas Munsch

**Affiliations:** 1 Institut für Physiologie, Otto-von-Guericke-Universität, Magdeburg, Germany; 2 Center for Behavioral Brain Sciences, Magdeburg, Germany; 3 Abteilung für Genetik & Molekulare Neurobiologie, Institut für Biologie, Otto-von-Guericke-Universität, Magdeburg, Germany; University of Cincinnatti, United States

## Abstract

The neuropeptide S (NPS) receptor system modulates neuronal circuit activity in the amygdala in conjunction with fear, anxiety and the expression and extinction of previously acquired fear memories. Using *in vitro* brain slice preparations of transgenic GAD67-GFP (Δneo) mice, we investigated the effects of NPS on neural activity in the lateral amygdala as a key region for the formation and extinction of fear memories. We are able to demonstrate that NPS augments excitatory glutamatergic synaptic input onto both projection neurons and interneurons of the lateral amygdala, resulting in enhanced spike activity of both types of cells. These effects were at least in part mediated by presynaptic mechanisms. In turn, inhibition of projection neurons by local interneurons was augmented by NPS, and subthreshold oscillations were strengthened, leading to their shift into the theta frequency range. These data suggest that the multifaceted effects of NPS on amygdaloid circuitry may shape behavior-related network activity patterns in the amygdala and reflect the peptide's potent activity in various forms of affective behavior and emotional memory.

## Introduction

The recently discovered NPS has received considerable attention as a modulator of neuronal and immunological functions [1], [2]. In fact, polymorphisms and splice variants of the cognate NPS receptor (NPSR) were recognized in conjunction with allergic diseases, immune responses, sleepiness, inflammatory bowel disease and panic disorder [3]–[7]. The NPSR was found to display high-affinity saturable and displaceable binding of NPS in the subnanomolar range [1], [8], and structure-activity and conformation-activity studies have identified key residues for biological activity of the receptor [9], [10]. In heterolog expression systems, NPS was shown to induce mobilization of intracellular Ca^2+^ and synthesis of cAMP, most likely by stimulating G_q_ and G_s_ [11], [12], suggesting that NPS may enhance cellular excitability [13].

In animal experiments, the NPS transmitter system has been implicated in arousal, fear and anxiety, energy and endocrine homeostasis, ethanol intake, sleep and locomotor activity [1], [14]–[17]. Of particular interest is the unique property of NPS to act both as an arousal-promoting and anxiolytic agent [11], [18], [19]. Consistent with the key role of the amygdala in these functions [20] and the expression of NPSR in the mouse lateral (LA) and basolateral (BLA) amygdala as well as neighboring endopiriform nucleus (EPN), studies on cellular NPS effects in the nervous system so far have focused on this structure. Jüngling and coworkers (2008) demonstrated that NPS via presynaptic NPSR on LA projection neurons enhances glutamatergic transmission onto GABAergic neurons of the intercalated cell mass of the amygdala, thereby facilitating extinction of auditory cued fear memories. Moreover, we [21] could previously show that NPS, via NPSRs in the EPN, alters the activity of both projection neurons and interneurons in the BLA, leading to a disturbed expression of contextual fear memory. These findings suggest a potential role of NPS in the interplay of amygdaloid circuits that mediate specific aspects of conditioned fear.

In the current study, we further investigated NPS effects on neuronal activity and subthreshold oscillations in the mouse LA, the primary sensory interface of the amygdala fear-conditioning circuitry [20], [22]–[25]. Applying slice physiology techniques to projection neurons and interneurons identified through a transgenic live fluorescence marker [26], we observed both direct and indirect NPS effects in the LA that culminated in a modulation of rhythmic cellular activities in the theta frequency range. Our data have implications for the understanding of divergent network processing in amygdala subnuclei and their integration through behaviorally relevant network activity patterns.

## Results

### NPS stimulates glutamatergic synaptic activity in LA projection neurons

**Figure 1:**
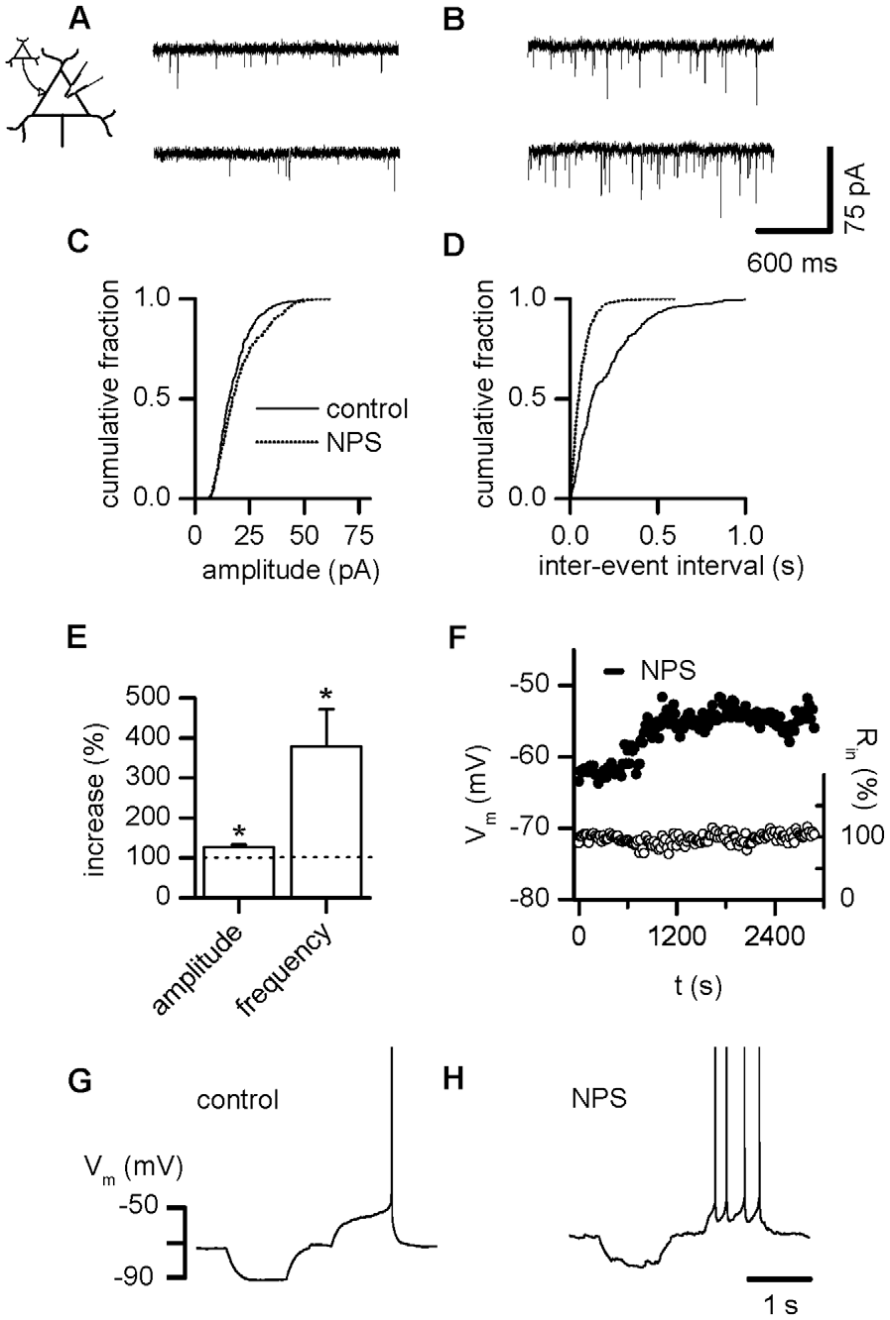
NPS stimulates glutamatergic input in LA projection neurons. (A) Examples of glutamatergic sEPSCs recorded in a LA projection neuron before and (B) during action of NPS. (C) Cumulative amplitude and (D) inter-event interval histograms obtained from the same neuron shown in (A, B) before addition of NPS and after a steady-state effect had been reached. (E) Normalized sEPSC amplitude and frequency pooled during control conditions and after addition of NPS demonstrates a significant increase in sEPSC amplitude as well as frequency. (F) Time course of NPS effect and input resistance for a representative PN. (G, H) Under current-clamp conditions, NPS application induces a depolarizing response associated with increased spike activity triggered upon depolarizing current injections in LA projection neurons. * P<0.05, ** P<0.01.

First, we determined the potential effect of exogenous NPS application on the activity of principle cells in the LA. We observed an increase of spontaneous EPSCs (sEPSCs) upon addition of 200 nM NPS. Recordings of sEPSCs were subjected first to the Kolmogorov-Smirnov test as a nonparametric test of equality of one-dimensional probability distributions used to compare two samples, the control sample and the NPS-treatment sample for each individual cell. NPS was considered effective when the increase reached p≤0.05, which was the case in 7 out of 9 projection neurons tested (Fig. 1).

In all cells, EPSCs were blocked to 99.6±0.4% (n = 5) in the presence of 10 µM 6,7-Dinitroquinoxaline-2,3-dione (DNQX) in combination with 50 µM DL-2-Amino-5-phosphono-pentanoic-acid (AP5), evidencing their mediation by glutamatergic AMPA and/or NMDA receptors. Typical current traces under control conditions (Fig. 1A) and during the presence of NPS (Fig. 1B) as well as cumulative amplitude (Fig. 1C) and inter-event interval (Fig. 1D) histograms illustrate the rise in amplitude as well as the shortening of inter-event intervals upon addition of NPS in a representative neuron. Normalized mean values for amplitude and frequency are shown in Fig. 1E. The average maximal amplitude of sEPSCs changed significantly from 15.8±2.3 pA in the absence to 20.6±3.9 pA in the presence of NPS, reflecting an increase to 126.8±6.6% (Fig. 1E, n = 7, p = 0.031). In parallel, a significant increase in average frequency from 7.0±2.2 Hz to 19.7±5.5 Hz was detected, yielding a rise to 379.9±91.8% (Fig. 1E, n = 7, p = 0.016).

Next, we tested NPS application upon sEPSCs recorded from projection neurons out of an isolated LA slice to check for the origin of NPS action. Part of the BA was left for improved mechanical handling. As basal amygdala (BA) neurons showed a complete absence of NPS-effects when separated from the endopiriform nucleus [21], interference due to the synaptic connectivity in between LA and BA seemed unlikely. Actually, in isolated slices, addition of 200 nM NPS still led to an increase of sEPSCs in 6 out of 8 projection neurons tested (data not shown). The average maximal frequency of sEPSCs changed significantly from 2.4±0.3 Hz in the absence to 6.4±1.1 Hz in the presence of NPS, reflecting an increase to 275.4±44.8% (n = 6, p = 0.031). Additionally, also the average maximal amplitude of sEPSCs changed significantly from 11.3±1.6 pA in the absence to 15.2±2.9 pA in the presence of NPS, reflecting an increase to 132.3±11.7% (n = 6, p = 0.031, data not shown). Changes of sEPSC amplitude or frequency induced by NPS, respectively, were not significantly different in neurons recorded from the intact or cut slice preparation (amplitude: p = 0.836, frequency: p = 0.445). Therefore, in contrast to NPS effects in the basal amygdala, the underlying mechanism of NPS action in projection neurons out of the LA seems to reside in the lateral amygdala itself.

Enhanced excitatory glutamatergic synaptic activity induced by NPS contributed to a membrane depolarization from resting membrane potential (–70.6±2.1 mV, n = 10) in 10 out of 11 projection neurons under current-clamp conditions, with an average maximal amplitude of 3.4±0.5 mV (n = 10), as shown for a representative PN, with no change in input resistance (Fig. 1F). Typical membrane potential responses to the current protocol composed of alternating negative and positive current pulses (−50 pA, +100 pA) are shown in figure 1G, H. NPS action was accompanied by an increase in presumably glutamatergic depolarizing synaptic events (Fig. 1H). The input membrane resistance amounted to 457.0±61.1 MΩ in the absence and 476.0±67.5 MΩ in the presence of NPS, remaining unaltered at 103.3±3.8% during NPS action (n = 10, p = 0.203). Mean spike frequency elicited by positive current injections adjusted to elicit one to three action potentials increased significantly from 2.0±0.6 Hz (n = 10) before addition of NPS to 4.6±0.9 Hz (n = 10, p = 0.002) during maximal drug action. Meanwhile, spike threshold remained unaltered in the presence of NPS (control: −42.7±0.9 mV, NPS: −42.4±0.9 mV, n = 9, p = 0.203).

**Figure 2:**
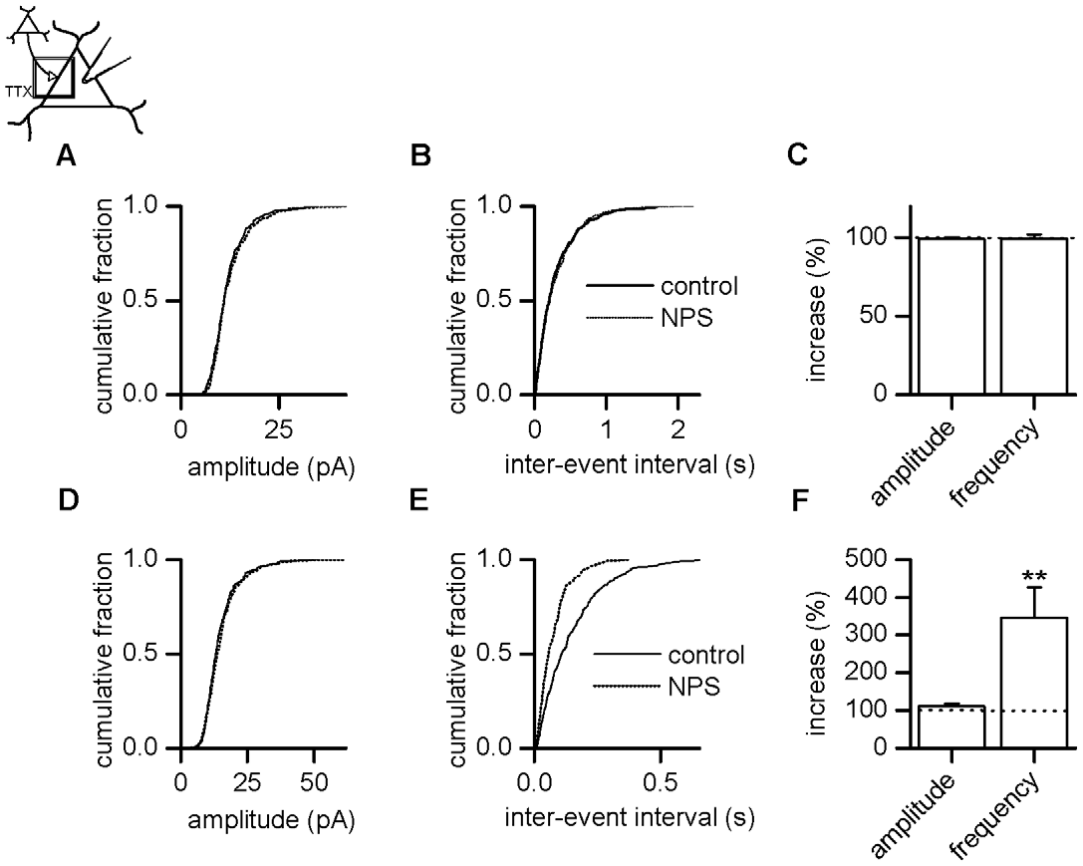
NPS increases mEPSCs frequency in a subpopulation of projection neurons. (A) Cumulative amplitude and (B) inter-event interval histograms obtained before and after NPS addition from a neuron showing no effect mediated by NPS. (C) In 9 out of 20 neurons, mEPSCs recorded in the presence of TTX were unchanged in amplitude as well as frequency by addition of NPS, as verified by the Kolmogorov-Smirnov test. (D) Cumulative amplitude and (E) inter-event interval histograms obtained from a neuron showing an increase in frequency after addition of NPS. (F) Normalized mEPSC amplitude and frequency pooled during control conditions and after addition of NPS demonstrates a significant increase in sEPSC frequency in a subpopulation of 11 out of 20 projection neurons. ** P<0.01.

In line of the recurrent network described in the LA [27], activation of postsynaptic NPS receptors on LA projection neurons followed by depolarization and action potential generation (see above) could give rise to the observed increase in sEPSCs. Nevertheless, NPS did not induce any postsynaptic current in voltage clamp in the presence of TTX (n = 4) or glutamatergic transmission blockers (n = 3) (data not shown). Furthermore, responses to exogenous glutamate were not affected by as much as 10 µM NPS in LA projection neurons in the same strain of mice [28], which also argues against a postsynaptic mechanism of NPS action. Thus, augmented sEPSCs in LA projection neurons may result from activation of NPS receptors located at glutamatergic presynaptic terminals, leading to an increase in intracellular Ca^2+^ as shown for cellular NPS action [11], and thereby facilitate glutamate release. In the presence of TTX to block action potential dependent transmitter release, amplitude and frequency of miniature EPSCs (mEPSCs) resolved two subpopulations as verified by the Kolmogorov-Smirnov test (see above). The first subpopulation was characterized by a lack of NPS effect upon amplitude as well as frequency of mEPSCs as illustrated as cumulative amplitude (Fig. 2A) and inter-event interval (Fig. 2B) histogram for a representative neuron. Mean mEPSC amplitude amounted to 9.8 pA±0.7 pA before and 9.7±0.7 pA (p = 0.301, n = 9) after addition of the peptide (Fig. 2C, n = 9, 99.3±1.0%). Frequency was unchanged by NPS application with values of 2.6±0.4 Hz before and 2.6±0.4 Hz after drug addition (p = 0.250, n = 9, 99.4±2.5%, Fig. 2C, see also Fig. S1B).

The second group of projection neurons is exemplified as cumulative amplitude (Fig. 2D) and inter-event interval (Fig. 2E) histograms for a representative neuron. mEPSC amplitude was likewise unaltered by NPS. Mean control amplitudes of 9.6 pA±0.9 pA (n = 11) resembled values in the presence of NPS of 10.9±1.4 pA (n = 11, p = 0.365, 111.1±7.2%, Fig. 2F). In contrast, frequency increased upon NPS addition from 2.9±0.5 Hz to 9.9±2.7 Hz (p = 0.001, n = 11, 345.6±81.4%, Fig. 2F, see also Fig. S1A). An increase in miniature EPSC frequency, but not amplitude, is consistent with a presynaptic mode of NPS action in the lateral amygdala.

### NPS stimulates glutamatergic synaptic activity in LA interneurons

Next, we characterized potential NPS effects onto LA local circuit interneurons, which are known to be involved in fear memory formation and extinction [29]. Mediation of sEPSCs by glutamatergic receptors in these cells was verified through selective blockage by DNQX (10 µM) and AP5 (50 µM) to 99.7±0.2% (n = 8).

**Figure 3:**
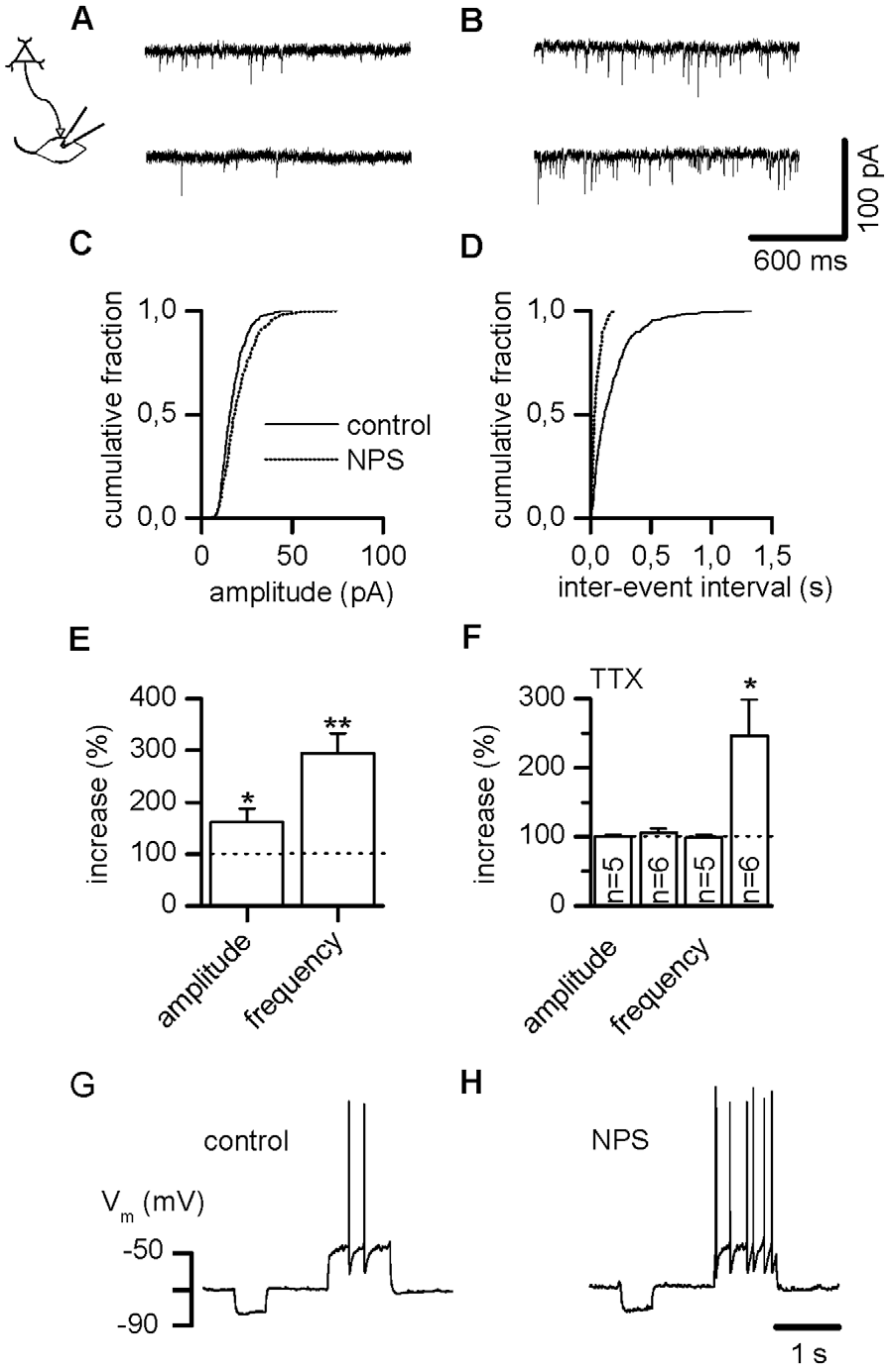
NPS stimulates glutamatergic input in LA interneurons. (A) Examples of glutamatergic sEPSCs recorded in a LA interneuron before and (B) during action of NPS. (C) Cumulative amplitude and (D) inter-event interval histograms obtained from the same neuron shown in (A, B) before addition of NPS and after a steady-state effect had been reached. (E) Normalized sEPSC amplitude and frequency pooled during control conditions and after addition of NPS demonstrates a significant increase in sEPSC amplitude as well as frequency. (F) mEPSCs recorded in the presence of TTX were unchanged in amplitude but exhibited augmented frequency in a subpopulation of neurons. (G, H) Under current-clamp conditions, NPS application induces a depolarizing response associated with increased spike activity triggered upon depolarizing current injections in LA interneurons. * P<0.05, ** P<0.01.

As found in projection neurons, modulation of sEPSCs by NPS was detected in 9 out of 10 interneurons. The effect is exemplified as current traces before (Fig 3A) and after (Fig 3B) application of the drug and summarized as cumulative amplitude (Fig. 3C) and inter-event interval (Fig. 3D) histograms for a representative neuron. Amplitudes rose significantly from 17.4±2.1 pA before to 29.4±7.0 pA after NPS application (n = 9, p = 0.020), while frequency increased significantly from 8.5±1.7 Hz to 24.5±5.5 Hz (n = 9, p = 0.004), respectively. Enhancement of normalized values averaged to 161.4±26.1% regarding amplitudes and 295.1±38.0% with respect to frequency (Fig. 3E, n = 9).

The site of action of this NPS effect was determined by analyzing mEPSCs. In the presence of TTX, frequency increased significantly in 6 out of 11 cells from 3.5±0.8 Hz to 6.9±1.0 Hz reflecting a rise to 246.6±52.3% (Fig. 3F, p = 0.031, see also Fig. S1C) but stayed constant in the remaining 5 interneurons. The latter displayed mean control frequencies of 4.6±0.7 Hz (n = 5), resembling values in the presence of NPS of 4.5±0.6 Hz, corresponding to 98.8±4.1% (Fig. 3F, n = 5, p = 0.625, see also Fig. S1D). Modulation of frequency in the presence of TTX points to a second presynaptic component of NPS action. In contrast, amplitudes of mEPSCs in interneurons under control and NPS condition were alike in all recorded interneurons, amounting to 10.4±1.1 pA and 10.8±0.8 pA (106.1±5.4%, Fig. 3F, n = 6, p = 0.438) in the responder group, and 10.8±1.8 pA and 10.9±2.0 pA (100.8±1.9%, Fig. 3F, n = 5, p = 0.875) in the non-responder group, respectively.

Excitation did lead to a depolarization under current clamp condition in 10 out of 13 interneurons averaging to 2.4±0.4 mV (n = 10) from the resting membrane potential of −71.5±1.4 mV (n = 10). Representative membrane potential responses to the current protocol composed of alternating negative and positive current pulses (−50 pA, +50 pA) are shown in figure 3G, H. The input membrane resistance remained unchanged before and after addition of NPS (control: 388.6±66.5 MΩ, NPS: 394.6±67.9 MΩ, 101.8±0.9%, n = 10, p = 0.203). Mean spike frequency elicited by positive current injections adjusted to elicit one to three spikes increased significantly from 2.6±0.8 Hz (n = 10) before addition of NPS to 9.7±2.1 Hz (n = 10, p = 0.002) during maximal drug action. Meanwhile, spike threshold remained unaltered in the presence of NPS (control: −43.8± 0.9 mV, NPS: −43.7±1.1 mV, n = 10, p = 0.734).

### NPS stimulates GABAergic synaptic activity in LA projection neurons

Projection neurons in the LA are subjected to strong inhibition through activation of local interneurons. As interneurons were excited by NPS (see above), we next characterized inhibitory synaptic transmission onto projection neurons. Mediation of sIPSCs by GABA_A_ receptors was verified through block by bicuculline (20 µM) to 99.1±0.3% (n = 8).

**Figure 4:**
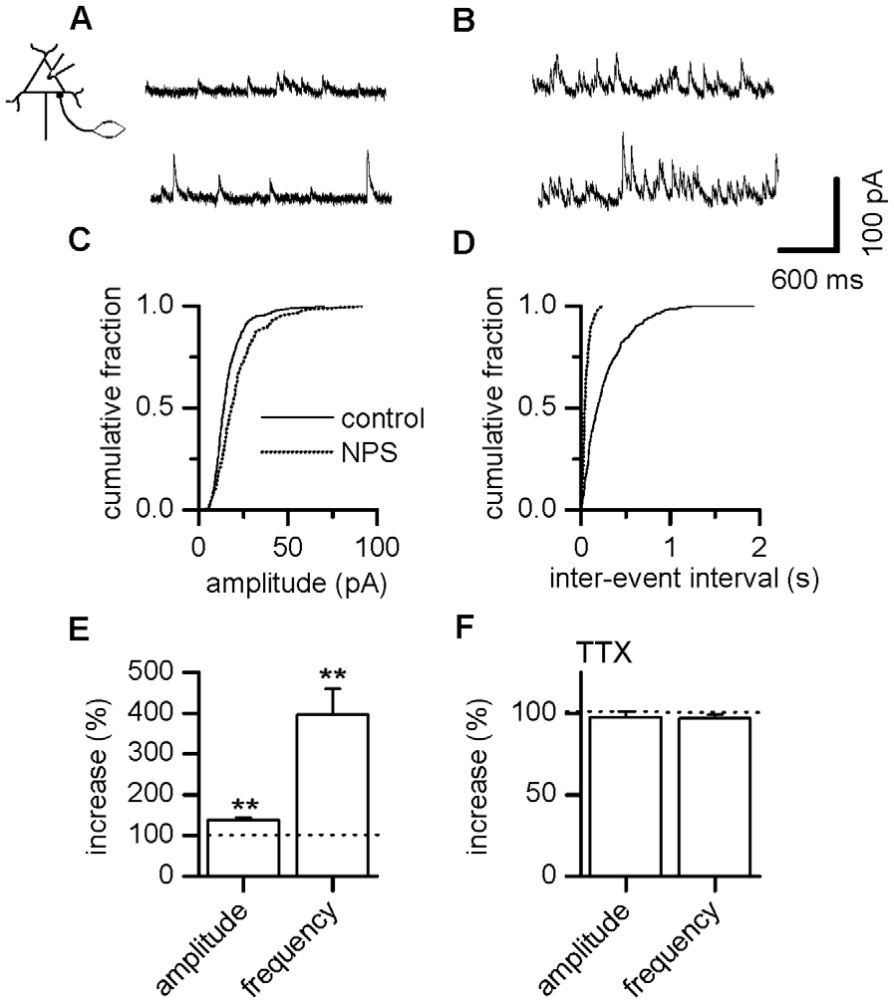
NPS stimulates GABAergic input in LA projection neurons. (A) Examples of GABAergic sIPSCs recorded in a LA projection neuron before and (B) during action of NPS. (C) Cumulative amplitude and (D) inter-event interval histograms obtained from the same neuron shown in (A, B) before addition of NPS and after a steady-state effect had been reached. (E) Normalized sIPSC amplitude and frequency pooled during control conditions and after addition of NPS exhibits a significant increase in sIPSC amplitude as well as frequency. (F) mIPSCs recorded in the presence of TTX were unchanged in amplitude as well as frequency by addition of NPS. * P<0.05, ** P<0.01.

The modulation of sIPSCs by NPS in 14 out of 15 neurons tested is exemplified as current traces before (Fig. 4A) and after (Fig. 4B) application of the drug and summarized as cumulative amplitude (Fig. 4C) and inter-event interval (Fig. 4D) histograms for a representative neuron. Mean amplitudes were changed significantly and amounted to 16.1±1.2 pA in the absence and 22.7±2.5 pA in the presence of NPS, yielding an increase to 138.0±5.7% (Fig. 4E, n = 14, p = 0.0001). In parallel, average frequency shifted significantly from 4.8±0.6 Hz to 14.8±1.2 Hz, accounting for an increase to 396.7±63.1% (Fig. 4E, n = 14, p = 0.0001).

This effect was not mediated by a mechanism corresponding to NPS receptor activation located at presynaptic terminals, as it was eliminated by block of action potentials (Fig. 4F). In the presence of TTX, amplitude as well as frequency of mIPSCs under control and NPS condition were alike, amounting to 14.6±1.1 pA and 14.4±1.4 pA (n = 5, p = 0.813) or 3.2±0.8 Hz and 3.1±0.8 Hz (n = 5, p = 0.250), resulting in normalized values of 97.5±3.8% and 97.0±2.2%, respectively (Fig. 4F).

Excitatory synaptic transmission is most likely prerequisite for the increase in GABA release seen in projection neurons. Indeed, blocking excitatory synaptic transmission by application of NBQX and AP5 abolished the effect on sIPSCs seen in projection neurons (data not shown). Mean control amplitudes of 14.2±0.7 pA (n = 5) resembled values in the presence of NPS of 13.8±0.8 pA (97.4±2.6%, n = 5, p = 0.375). Likewise, frequency averaged to 6.5±1.6 Hz before and 6.2±1.4 Hz after addition of NPS (95.9±1.4%, n = 5, p = 0.063).

### NPS triggers action potentials in LA projection neurons as well as interneurons

**Figure 5:**
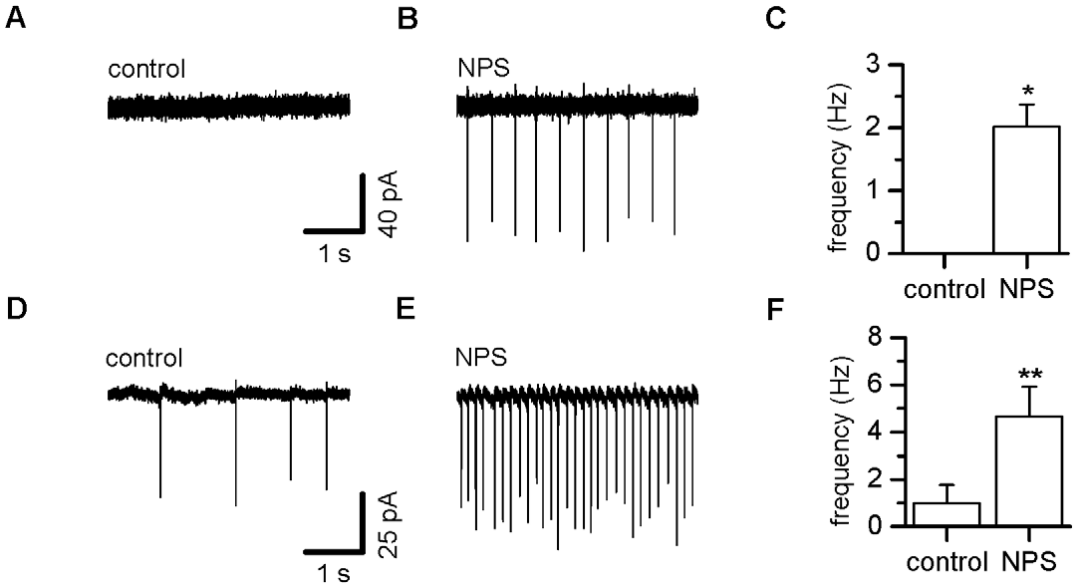
NPS increases frequency of action potential currents recorded using cell-attached voltage-clamp. (A) Baseline trace before and (B) after addition of NPS in a representative projection neuron displays an increase in downward vertical deflections indicating spike currents. (C) Summary of NPS effects on spike activity pooled for all tested projection neurons (n = 5). (D) Control trace before and (E) after addition of NPS in a representative interneuron also shows increased action potential activity, as pooled (F) for all recorded interneurons (n = 4). * P<0.05, ** P<0.01.

The difference in IPSC frequency with/without TTX suggests that some interneurons in the lateral amygdala are spiking in the presence of NPS. Moreover, the fact that the change in sIPSCs is abolished by NBQX and AP5 indicates that projection neurons in the lateral amygdala also must fire action potentials after NPS addition. Cell-attached recordings from projection neurons and interneurons in the loose seal configuration are shown in Figure 5. Indeed, projection neurons (Fig. 5A, B, C) as well as interneurons (Fig. 5D, E, F) displayed enhanced spike activity when NPS was added. Effects are exemplified as current traces before (Fig 5A, D) and after (Fig 5B, E) application of NPS and summarized in Fig. 5C, F. In projection neurons, spike frequency rose from 0 Hz to 2.0±0.4 Hz (n = 5, p = 0.005), while interneurons displayed frequencies of 1.0±0.8 Hz before and 4.7±1.2 Hz (n = 4) after drug addition (p = 0.04). In two out of five PNs, firing triggered by NPS showed regular inter-spike intervals varying by 1.9 and 1.4 Hz, respectively. Scatter in between maximal and minimal instantaneous frequency ranged from 9.9 to 1.4 Hz, mean values amounted to 5.3±1.6 Hz (n = 5). In interneurones, one out of four cells revealed a rhythmic firing pattern after addition of NPS. Variation reached from 6.3 to 15.1 Hz with a mean value of 10.2±1.9 Hz (n = 4).

### NPS stimulates subthreshold oscillations in LA projection neurons

LA projection neurons generate stable subthreshold membrane potential oscillations at around 2.4 Hz [30], which are implicated in shaping network activity related to fear and anxiety [31]. As NPS is a modulator of some aspects of fear and extinction, we examined oscillatory activity on the LA in the presence of NPS.

**Figure 6:**
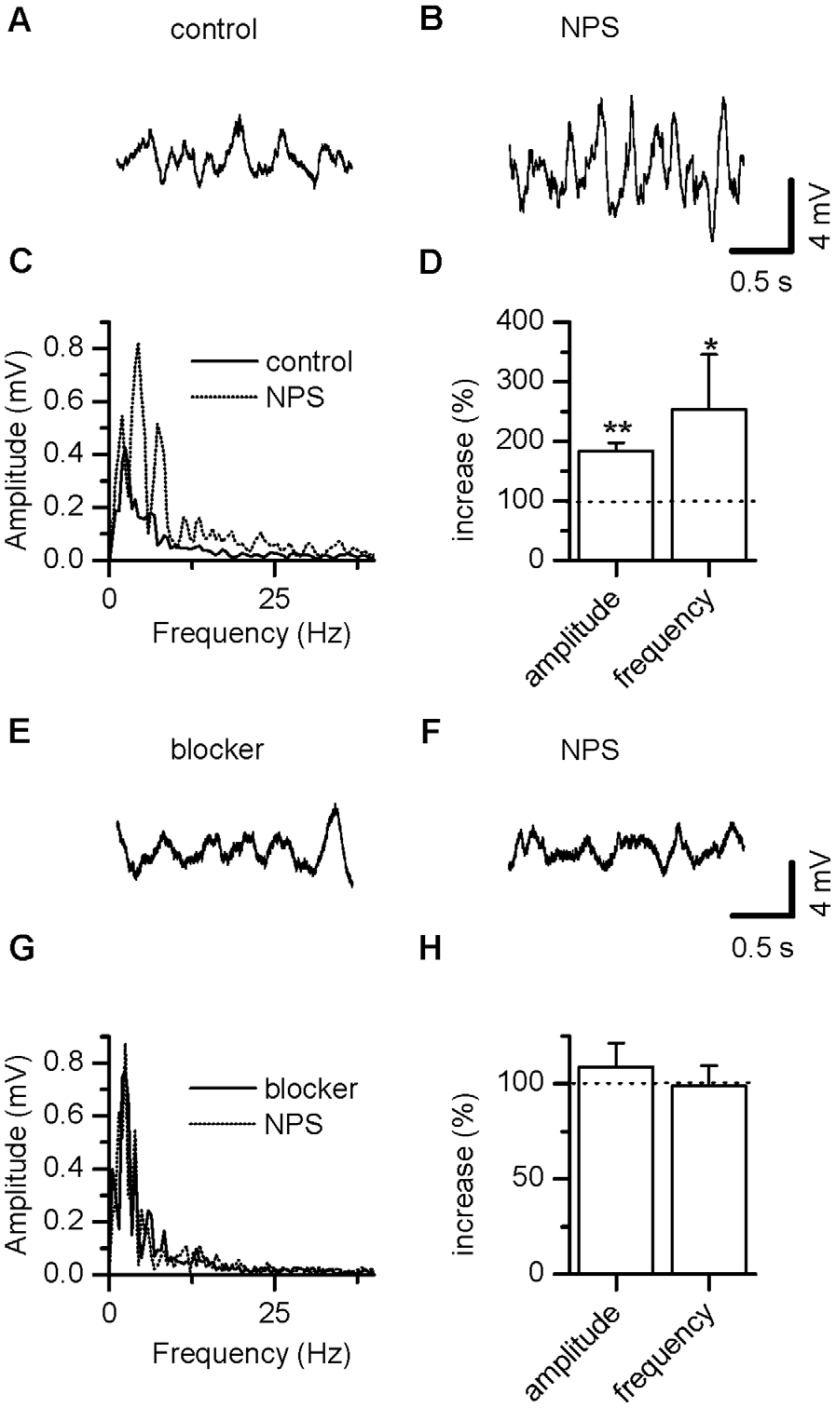
NPS enhances subthreshold membrane potential oscillations in LA projection neurons. (A, B) Examples of subthreshold oscillations in a LA projection neuron before (A) and during (B) action of NPS. (C) Corresponding FFT analysis illustrate predominant theta frequencies enhanced by NPS. (D) Peak amplitude as well as frequency are significantly enlarged by addition of NPS. (E, F) Examples of subthreshold oscillations in a LA projection neuron before (E) and during (F) action of NPS in the presence of AP-5 and DNQX to block glutamatergic transmission. (G) Corresponding FFT analysis illustrate block of NPS effect. (H) Peak amplitude as well as frequency are unchanged by NPS * P<0.05, ** P<0.01.

Figure 6A shows a representative example of subthreshold membrane potential oscillations upon membrane depolarization by a steady current injection (85 pA, duration 8 s) in an LA projection neuron. After application of NPS (100 nM), oscillations were enhanced (Fig. 6B). Fast Fourier Transformation (FFT) demonstrates the rhythmic nature of the membrane potential deflections (Fig. 6C). In the presence of NPS, subthreshold oscillation frequency rose significantly from 2.6±0.5 Hz to 4.3±0.3 Hz (n = 8, p = 0.016) yielding an increase of 253.8±92.1% after addition of NPS. Concurrently, peak amplitude changed significantly from 0.6±0.1 mV to 1.0±0.2 mV (n = 8, p = 0.008) in the presence of NPS, as calculated to 183.8±13.9% (Fig. 6D). In the presence of AP-5 and DNQX, subthreshold oscillations were not significantly altered as compared to control (frequency: 2.1±0.3, n = 10, p = 0.274; peak amplitude: 0.6±0.08, p = 0.360). Meanwhile, NPS was ineffective under these conditions (frequency: 2.0±0.3, n = 10, p = 0.438; peak amplitude: 0.7±0.08, p = 0.813, Fig, 6E–H). This suggests that NPS action upon subthreshold oscillations is mediated by its enhancing effects on glutamatergic transmission.

## Discussion

In the present study we show that NPS is a potent modulator of glutamatergic transmission onto both projection neurons and local circuit interneurons in the lateral amygdala and thus capable of controlling the activity and rhythmicity of LA principle cells. This adds to the manifold NPS effects onto amygdaloid circuitry and bears functional implications, in particular, for fear related information processing and the control of anxiety states in this structure.

As a key observation in our experiments, we recorded an increase in spontaneous excitatory synaptic transmission in neurons of the LA upon addition of 200 nM NPS. Under current clamp conditions, this resulted in an enhancement of projection neuron spiking activity. Presumably, this response arose from activation of the NPSR on presynaptic terminals in the LA itself, as indicated by the increase in frequency, but not amplitude, of miniature EPSCs in a subset of projection neurons. In addition, postsynaptic effects of NPS were not observed in the present study, and are therefore unlikely to contribute substantially to the increase in spontaneous excitatory synaptic transmission.

NPS-activated synapses may comprise terminals of local projections within the LA [27] and/or inputs to the LA [32]. The restricted mRNA expression pattern of the NPSR, however, suggests that the effect on potential afferent synapses would be limited to specific afferent inputs, such as those arising from the EPN [33]. On the other hand, LA projection neurons also express the NPSR. Presynaptic NPS receptors have been located to terminals of LA principle neurons and were shown to modulate glutamatergic transmission onto neurons of the intercalated cell mass [28].

As described for projection neurons of the basal amygdala [34], a segregation of distinct neuronal circuits related to anatomical connectivity in otherwise intermingled and akin neurons might also give rise to distinct populations of projection neurons. Along this line, the two populations of NPS-responsive and non-responsive neurons observed in this study may indeed be characterized by quantitative and qualitative differences in their afferent glutamatergic terminals. While around half of neurons demonstrated NPS-activated presynapses, most recorded PNs showed increases in sEPSCs, spike activity and oscillations. This may relate to the fact that a subpopulation of responding neurons may trigger widespread changes in network activity.

Similar to its effect on projection neurons, we observed an increase of sEPSCs through NPS also in LA interneurons (Fig. 2). As a strong feedback inhibition controls principal cell activity in the amygdala [35], the observed excitation of interneurons may to a large extend be attributable to an indirect effect of NPS resulting from the excitation of LA projection neurons. Interestingly, we observed this excitation only in a subpopulation of LA interneurons.

Various populations of interneurons exist in the LA, which differ in respect to their morphology, firing patterns and their involvement in feed-forward and feed back circuitries [36]. Moreover, evidence suggests the existence of at least two distinct types of projection neurons that vary in their relation with interneurons [37]. In the NPS-responsive subpopulation of interneurons we observed effects that were comparable to those on LA projection neurons, with a persistent modulation of mEPSCs frequency in the presence of TTX. Additional evidence for this scenario was provided by cell-attached recordings, which are adequate for recording action potential currents in voltage-clamp mode without changing the firing activity of the cell [38]. Under these recording conditions both types of LA neurons showed a substantial increase in action potential frequency after addition of NPS.

 Taken together, the modulation of local circuit activity through NPS is largely reminiscent of this neuropeptidés effects in the BA [21], with two important exceptions: Firstly, NPS effects seem to be more uniform in the lateral amygdala, as most (10 out of 11) LA projection neurons, but only a subset of BA projection neurons, showed a robust increase of spike activity. And secondly, direct presynaptic NPS effects were only observed in LA, but not in BA neurons. We have previously demonstrated that in the BA neural activity is shaped via the neighboring endopiriform nucleus (EPN). Direct projections from the EPN to all deep amygdaloid nuclei including the LA have been described [39], suggesting that a similar mechanism might also work towards the LA. However, our data show that NPS modulation of neural activity in the LA, in contrast to the BA, can occur in the absence of the EPN.

Our findings are in good agreement with the expression of the NPSR mRNA in the mouse EPN, LA, and BA [28]. Interestingly, in the rat, expression is observed in the EPN and intercalated cells, but only scarcely in the LA and BA [33], indicating that a common feature of NPS function in these species may be related to the modulation of EPN-mediated input to the LA/BA and its output towards the central amygdala. This is in line with the rather selective effects of the peptide on contextual fear memory and the (highly context-dependent) extinction of previously conditioned fear [21], [28]. Moreover, it appears that direct NPS actions are restricted to regions that primarily receive unimodal sensory input (the EPN - olfactory, the LA - auditory, visual), whereas regions receiving polymodal input such as the BA (hippocampal, entorhinal cortex) or intercalated cells (prefrontal cortex) are only indirectly modulated through NPS.

Given the high expression of NPSR and the prominent physiological effects of NPS in the LA, it is striking that the strongly LA-dependent acquisition of auditory cued fear memories remains unaffected by NPS [28]. As a possible explanation for this, we hypothesize that NPS effects may only become behaviorally relevant during and following fear memory recall. Rhythmic network activities are thought to play an important role in these stages, through binding stimulus-specific, contextual and operant aspects of fear memory [40]–[42]. Within this context it is interesting that LA projection neurons can generate slow oscillations of the membrane potential through an interplay of intrinsic membrane conductance [30].

The ability of generating intrinsic membrane potential oscillations is hypothesized to endow LA cells with the capacity to behave like unitary oscillators that can exhibit resonant behavior. Thereby, subthreshold oscillations can synchronize synaptic input signals [43] to promote population activity at preferred frequencies [44]. In addition, rhythmic inhibitory synaptic inputs can render the activity of neuronal populations to become synchronized [45]. In fact, both theoretical and experimental evidence suggest that interneurons support the timing and synchronization of oscillatory activity in neuronal networks [46]. It is conceivable that these processes are involved in the recruitment of amygdala activities to hippocampus-driven theta oscillation during fear memory retrieval [47]. Our current observations reveal that the slow-rhythmic intrinsic activity of LA projection neurons can be reinforced through neuromodulatory inputs like NPS, enhancing oscillations in the lower theta frequency band (3–6 Hz) with little effects on firing rates but providing time windows for synchronizing LA neuronal activity with afferent inputs.

These effects may result from the enhanced excitatory glutamatergic activity in the LA or modulation of voltage-dependent ionic conductance via NPS-responsive second messenger cascades [30]. In vivo, NPS released from stress-responsive afferents to the amygdala originating in the parabrachial nucleus and the locus coeruleus [11] may hence contribute to network activity patterns induced by different sensory modalities in the EPN and LA. On this basis NPS could support the integration of higher polymodal information concerning context and operation in the BA and/or the selective activation of different subpopulations of interneurons of the intercalated cell mass involved in fear memory renewal and extinction [34], [32].

## Materials and Methods

### Slice preparation

All experiments were carried out in accordance with the European Committees Council Directive (86/609/EEC) and approved by the local animal care committee (Landesverwaltungsamt Sachen-Anhalt). Juvenile (P12–P22) GAD67-GFP (Δneo) mice (Tamamaki et al., 2003) of either sex were decapitated after deep anesthesia with forene (isofluran, 1-Chloro-2,2,2-trifluoroethyl-difluoromethylether). Part of the brain including the amygdala was rapidly removed and transferred into chilled oxygenated saline of the following composition (mM): KCl, 2.4; MgSO_4_, 10; CaCl_2_, 0.5; piperazine-N,Ń'-bis(ethanesulphonic acid) (PIPES), 20; glucose, 10; sucrose, 195 (pH 7.35). Coronal slices (250 µm thick) were cut using a vibratome (Model 1000, The Vibratome Company, St. Louis, USA), and were incubated in standard artificial cerebrospinal fluid (ACFS) containing (in mM): NaCl, 120; KCl, 2.5; NaH_2_PO_4_, 1.25; NaHCO_3_, 22; MgSO_4_, 2; CaCl_2_, 2; glucose, 10; bubbled with 95%O_2_/5% CO_2_ to a final pH of 7.3. Single slices were then placed in a submersion chamber.

In some experiments, the LA was mechanically dissected under a binocular microscope by cutting off all tissue around the LA, leaving some parts of the basal amygdala for mechanical handling.

### Recording techniques

Recordings were performed in the whole-cell mode on lateral amygdala neurons using a patch-clamp amplifier (EPC-9, Heka, Lamprecht, Germany) under visual control by use of infrared videomicroscopy (S/W-camera CF8/1, Kappa, Gleichen, Germany) as described previously (Meis et al., 2008). A monochromator (Polychrome II, Till Photonics, Martinsried, Germany) connected to an epifluorescence system and a 40x/0.80 water immersion lens was used to identify neurons as interneurons by EGFP fluorescence. Projection neurons were identified by lack of fluorescence, as well as pyramidal-like morphology and spike frequency adaptation in response to prolonged depolarization [21]. Patch pipettes were pulled from borosilicate glass (GC150T-10, Clark Electromedical Instruments, Pangbourne, UK) to resistances of 2-3 MΩ. A liquid junction potential of 10 mV of the pipette solution was corrected for. For recordings of inhibitory postsynaptic currents (IPSCs), the pipette solution contained (in mM): Csgluconate, 117; CsCl, 13; MgCl_2_, 1; CaCl_2_, 0.07; EGTA, 11; HEPES, 10; MgATP, 3, NaGTP, 0.5 (pH 7.2 with KOH). Excitatory postsynaptic currents (EPSCs) were measured using an intracellular solution composed of (in mM): Kgluconate, 95; K_3_citrate, 20; NaCl, 10; HEPES, 10; MgCl_2_, 1; CaCl_2_, 0.1; EGTA, 1.1; MgATP, 3; NaGTP 0.5, (-)-bicuculline methiodide, 0.01 (pH 7.2 with KOH). Miniature postsynaptic currents (mIPSCs, mEPSCs) were isolated in the presence of 1 µM tetrodotoxin (TTX). After obtaining the whole cell configuration, neurons were held routinely at -70 mV for EPSCs or at 0 mV for IPSCs, respectively. For collecting current clamp data, pipettes were filled with (in mM): Kgluconate, 95; K_3_citrate, 20; NaCl, 10; HEPES, 10; MgCl_2_, 1; CaCl_2_, 0.1; EGTA, 1.1; MgATP, 3; NaGTP 0.5 (pH 7.2 with KOH).

Cell attached recordings were done in voltage-clamp mode with loose seals in between 10 - 20 MΩ. Pipettes were filled with ACSF and had resistances of approximately 3 MΩ. To avoid a change in firing activity of the cell due to stimulation, current measured by the amplifier (I_amp_) was kept at 0 pA [38].

### Data analysis

Miniature postsynaptic currents were detected using the program ‘Mini-Analysis’ (Jaejin software, Leonia, NJ, USA). Cumulative histograms without bins were calculated within time periods of 30 s to 3 min duration containing at least 300 events before addition and whilst maximal effect of NPS. Input resistance was quantified from the steady-state voltage deflection upon injection of small hyperpolarizing currents under current clamp conditions. Statistical analysis was performed using Kolmogorov-Smirnoff (Mini-Analysis) and nonparametric tests by Graph Pad Prism software (San Diego, CA, USA; Wilcoxon signed rank test for paired observations, Mann Whitney test for non paired observations). Data are presented as mean ± SEM. Differences were considered statistically significant at p≤0.05.

### Drugs

As recovery from responses to NPS could not be obtained with washout up to 1 hour (see also Figure 1F), the substance was applied only once to each slice. Drugs were added to the external ACFS. All substances were obtained from Sigma (Diesenhofen, Germany), except for NPS (Phoenix Europe GmbH, Karlsruhe, Germany), DNQX (Tocris, Bristol, UK), and TTX (Alomone, Jerusalem, Israel).

## Supporting Information

Figure S1 **Histogram of mEPSCs of NPS-responding and non responding neurons.**
mEPSC frequency of NPS-responders (A) and non-responders (B) of projection neurons is clearly shifted to larger values in the “responder group” after addition of NPS, whereas non-responders showed only little change from baseline. (C, D) Histogram of mEPSC frequency of NPS-responders and non-responders of interneurons. Bin size was 2Hz.
(TIF)
