# Supplementary figures and images for: Neuropeptide S-Mediated Facilitation of Synaptic Transmission Enforces Subthreshold Theta Oscillations within the Lateral Amygdala
Source: PLoS One. 2011 Mar 18;6(3):e18020. doi: 10.1371/journal.pone.0018020 (PMC3060922; doi:10.1371/journal.pone.0018020)

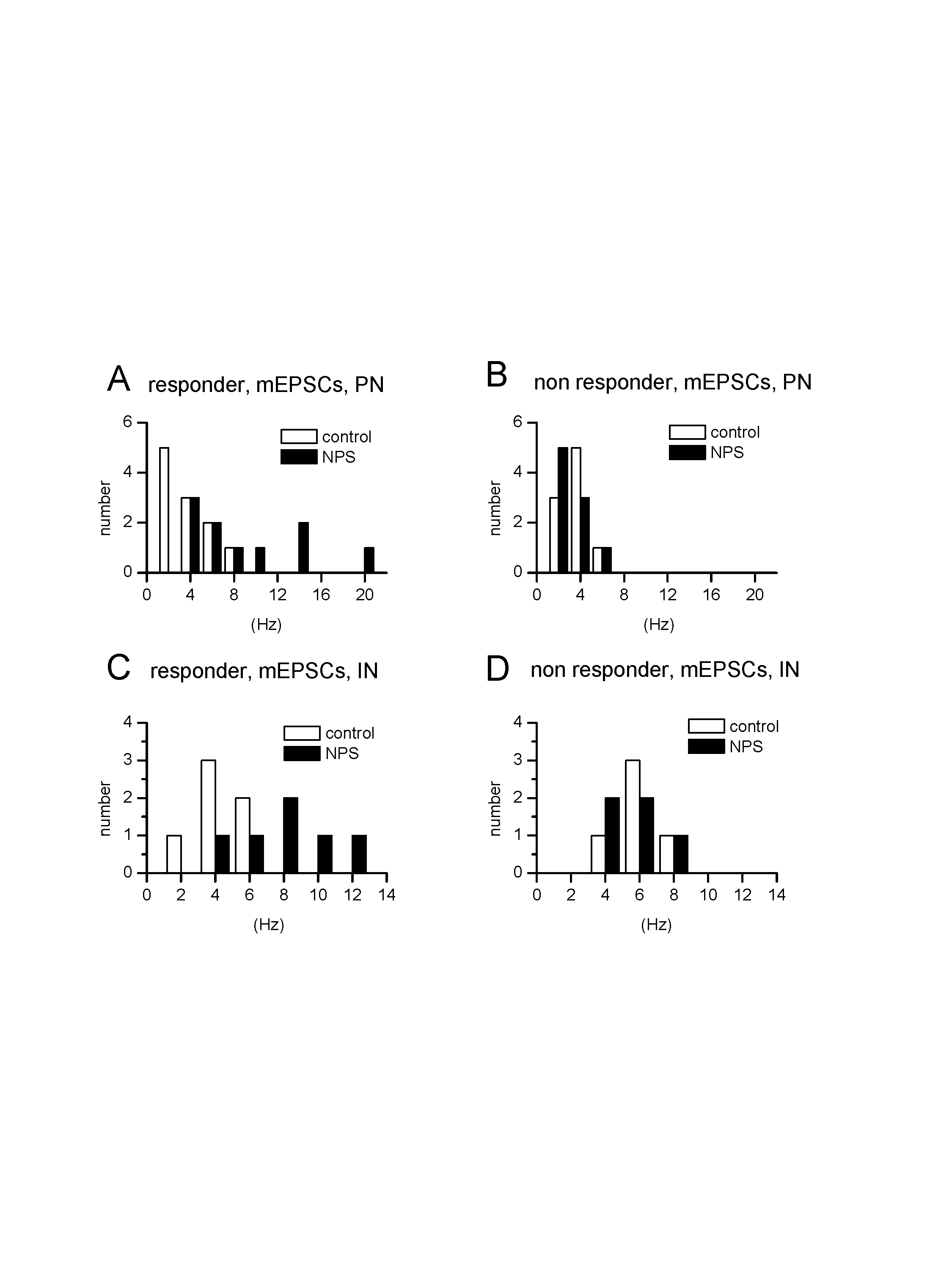

Supplement: Figure S1 — Histogram of mEPSCs of NPS-responding and non responding neurons. mEPSC frequency of NPS-responders (A) and non-responders (B) of projection neurons is clearly shifted to larger values in the “responder group” after addition of NPS, whereas non-responders showed only little change from baseline. (C, D) Histogram of mEPSC frequency of NPS-responders and non-responders of interneurons. Bin size was 2Hz. (TIF) [file pone.0018020.s001.tif]
